# Exploring TSPAN4 promoter methylation as a diagnostic biomarker for tuberculosis

**DOI:** 10.3389/fgene.2024.1380828

**Published:** 2024-04-12

**Authors:** Jiahao Zhang, Jilong Chen, Yan Zhang, Liuchi Chen, Weiwei Mo, Qianting Yang, Mingxia Zhang, Haiying Liu

**Affiliations:** ^1^ National Institute of Pathogen Biology, Chinese Academy of Medical Sciences & Peking Union Medical College, Beijing, China; ^2^ State Key Laboratory of Respiratory Health and Multimorbidity, NHC Key Laboratory of Systems Biology of Pathogens, National Institute of Pathogen Biology, Chinese Academy of Medical Sciences and Peking Union Medical College, Beijing, China; ^3^ National Institute of Pathogen Biology and Center for Tuberculosis Research, Chinese Academy of Medical Sciences and Peking Union Medical College, Beijing, China; ^4^ Shenzhen Clinical Research Center for Tuberculosis, Shenzhen, China; ^5^ National Clinical Research Center for Infectious Disease, Shenzhen Third People’s Hospital, The Second Affiliated Hospital, School of Medicine, Institute for Hepatology, Southern University of Science and Technology, Shenzhen, China

**Keywords:** tuberculosis, DNA methylation biomarkers, diagnosis, machine learning, qMSP

## Abstract

**Background:**

Tuberculosis (TB), caused by *Mycobacterium tuberculosis* (Mtb), is a persistent infectious disease threatening human health. The existing diagnostic methods still have significant shortcomings, including a low positivity rate in pathogen-based diagnoses and the inability of immunological diagnostics to detect active TB. Hence, it is urgent to develop new techniques to detect TB more accurate and earlier. This research aims to scrutinize and authenticate DNA methylation markers suitable for tuberculosis diagnosis. Concurrently, Providing a new approach for tuberculosis diagnosis.

**Methods:**

Blood samples from patients with newly diagnosed tuberculosis and healthy controls (HC) were utilized in this study. Examining methylation microarray data from 40 whole blood samples (22TB + 18HC), we employed two procedures: signature gene methylated position analysis and signature region methylated position analysis to pinpoint distinctive methylated positions. Based on the screening results, diagnostic classifiers are constructed through machine learning, and validation was conducted through pyrosequencing in a separate queue (22TB + 18HC). Culminating in the development of a new tuberculosis diagnostic method via quantitative real-time methylation specific PCR (qMSP).

**Results:**

The combination of the two procedures revealed a total of 10 methylated positions, all of which were located in the promoter region. These 10 signature methylated positions facilitated the construction of a diagnostic classifier, exhibiting robust diagnostic accuracy in both cross-validation and external test sets. The LDA model demonstrated the best classification performance, achieving an AUC of 0.83, specificity of 0.8, and sensitivity of 0.86 on the external test set. Furthermore, the validation of signature methylated positions through pyrosequencing demonstrated high agreement with screening outcomes. Additionally, qMSP detection of 2 potential hypomethylated positions (cg04552852 and cg12464638) exhibited promising results, yielding an AUC of 0.794, specificity of 0.720, and sensitivity of 0.816.

**Conclusion:**

Our study demonstrates that the validated signature methylated positions through pyrosequencing emerge as plausible biomarkers for tuberculosis diagnosis. The specific methylation markers in the *TSPAN4* gene, identified in whole blood samples, hold promise for improving tuberculosis diagnosis. This approach could significantly enhance diagnostic accuracy and speed, offering a new avenue for early detection and treatment.

## 1 Introduction

Tuberculosis is a persistent infectious ailment arising from Mtb infection. It is estimated that in 2022, globally, 10.6 million people (95% UI: 9.9–11.4 million) suffer from tuberculosis. In the same year, 7.5 million new cases of tuberculosis were diagnosed worldwide, resulting in 1.3 million fatalities. The estimated global incidence of tuberculosis in 2022 was 133 new cases per 100,000 population (95% UI: 124–143). The net reduction in the global number of deaths caused by TB from 2015 to 2022 was only 19%, far from the WHO End TB Strategy milestone of a 75% reduction by 2025. Swift identification and diagnosis of Mtb infection can effectively curb tuberculosis transmission. Presently, there exists a substantial disparity between the reported tuberculosis cases and the estimated total of tuberculosis infections, indicating the inadequacy of tuberculosis diagnosis. Based on the annual decline rate of the total number of existing cases, achieving the WHO End TB target by 2035 is extremely challenging without new technologies.

In recent years, despite progress in tuberculosis diagnosis, more than a third of clinical tuberculosis cases still lack effective and prompt identification. There is an urgent need to discover biomarkers for a more sensitive and earlier diagnosis of tuberculosis. Pathogen-based diagnosis, in use for a century, remains the established method for tuberculosis diagnosis. Despite rapid advancements in molecular pathogen-based diagnostic techniques, the positive rate of such diagnosis in tuberculosis patients is only around 60%, causing substantial diagnostic delays and contributing to social transmission. An increasing number of laboratories are turning their focus towards selecting and applying host diagnostic markers. However, the current emphasis is predominantly on the development of RNA and protein biomarkers (McNerney et al., 2012; Guo et al., 2022; Nogueira et al., 2022). Yet, the instability of RNA and the requirement for antigen stimulation in protein diagnosis often lead to diagnoses at later stages, thus limiting diagnostic effectiveness. Recognizing that DNA serves as the most upstream regulator, any disease-related changes in DNA can be leveraged for early diagnosis (Ziegler et al., 2012).

In recent years, DNA methylation has gained popularity in the realm of disease diagnosis. DNA methylation modifications can either silence or activate the expression of pertinent host genes (Baylin and Jones, 2011; Dawson and Kouzarides, 2012). Abnormal methylation modifications in the genome occur at CpG sites in early disease promoter regions, making them valuable for early diagnosis. In addition, numerous studies focus on the area adjoining the transcription start site (TSS) of the promoter. Furthermore, compared to other biomarkers, DNA methylation markers exhibit higher stability and are detectable in blood (Portela and Esteller, 2010; Ziegler et al., 2012; Papanicolau-Sengos and Aldape, 2022). For instance, in lung cancer patients, the methylation of SHOX2/PTGER4/RASSF1A in plasma DNA has been employed as a biological marker for identifying lung cancer and has found practical applications in clinical settings (Kneip et al., 2011; Weiss et al., 2017; Malpeli et al., 2019). Beyond lung cancer, methylation detection technology has found widespread use in clinically diagnosing various solid tumors (Han et al., 2019; Tang et al., 2019; Papanicolau-Sengos and Aldape, 2022; Chang et al., 2023). With the advancement of computational biology, several studies have utilized DNA methylation microarrays or methylation profiles to develop machine learning diagnostic classifiers. These classifiers can assist in disease diagnosis and have demonstrated effective diagnostic outcomes (Hao et al., 2017; Capper et al., 2018; Li et al., 2022b). Regarding infectious diseases, DNA methylation has exhibited substantial diagnostic potential in the diagnosis of chronic hepatitis B and cirrhosis patients (Zhao et al., 2014). Additionally, the methylation of the RASSF1A and TIMP3 promoter regions can be utilized to diagnose S. haematobium infection (Zhong et al., 2013). This undeniably underscores the immense potential of DNA methylation as a biomarker for disease diagnosis.

In the domain of tuberculosis diagnosis, there is a scarcity of research on DNA methylation biomarkers, and existing studies either lack methylation microarray analysis or concentrate solely on specific cells. The prevalent methods employed, such as Next-Generation sequencing, possess evident limitations for clinical application (Chen et al., 2014; Lyu et al., 2022). Presently, there are no widely applicable DNA methylation biomarkers or diagnostic methods for tuberculosis in clinical practice. Hence, this study is centered on screening and validating clinical samples for DNA methylation biomarkers associated with tuberculosis. The objective is to authenticate potential methylated positions using pyrosequencing, devise qMSP methods for their detection, and formulate potential tuberculosis diagnostic biomarkers alongside novel diagnostic methods suitable for clinical implementation.

## 2 Materials and methods

### 2.1 Study subjects

Clinical samples were collected from the Third People’s Hospital of Shenzhen City. In the TB group, inclusion criteria were positive results in pathogen diagnosis (sputum smear, bacterial culture or GeneXpert MTB/RIF) or immunological diagnosis (γ-interferon release assay). All patients were initially diagnosed with TB and received anti-tuberculosis drug treatment for less than 7 days. Patients with pneumonia, pulmonary fungal infections, HIV and HBV infections were excluded by clinical CT imaging, blood testing. For the HC group, inclusion criteria were a negative γ-interferon release assay, no clinical symptoms related to tuberculosis, no previous history of tuberculosis, and normal chest X-ray findings. DNA extraction from whole blood samples was performed using the QIAamp DNA Blood Mini Kit (Qiagen, Hilden, Germany), and DNA concentration was quantified with a spectrophotometer. The samples were stored at −80°C until use.

The study design was conducted in accordance with the principles of the Declaration of Helsinki and approved by the National Institute of Pathogen Biology, Chinese Academy of Medical Sciences and Peking Union Medical College (ethical approval number: IPB-2017-1).

### 2.2 Acquisition and processing of raw data

Firstly, we integrated 22 methylation microarray data from our laboratory (10TB + 12HC) with the methylation microarray data from GSE118469 (12TB + 10HC) to form the training set. Secondly, This training set, together with the gene chip GSE83456, was employed to identify signature methylated positions. Finally, The training set was used to train machine learning models, and the methylation microarray data GSE145714 (7TB + 12HC) was utilized as an external test set to evaluate the generalization capability of the machine learning models.

The methylation microarray data from 22 samples in the training set were acquired using the Illumina HumanMethylation450 BeadChip in our laboratory. Gene chip dataset GSE83456, GSE118469 in methylation microarray training set, and external test set GSE145714 were obtained from the Gene Expression Omnibus (GEO) database. All samples utilized in the aforementioned analysis must adhere to the inclusion criteria for TB and HC. Detailed dataset information is available in Supplementary Table S1. Raw data analysis involved the R package “ChAMP” for data import, batch effect processing, pre-processing, filtering, and differential and enrichment analysis (Morris et al., 2014; Tian et al., 2017). Enrichment analysis visualization was conducted using the R packages “Circlize” and “ComplexHeatmap” (Gu et al., 2014; Gu et al., 2016). Gene chip raw data import, pre-processing, and differential analysis were performed using the R package “limma” (Ritchie et al., 2015). Selection criteria for DMPs were Padjust <0.05, |Δβ| > 0.1; selection criteria for DMRs were *p*-value <0.025, minpositions >7, and maxgap <200; and selection criteria for DEGs were Padjust <0.05, |logFC| > 0.5.

### 2.3 Identification of signature genes

The WGCNA package was employed for sample clustering (Langfelder and Horvath, 2008). Following outlier removal, the adjacency matrix was transformed into a topological overlap matrix (TOM). Subsequently, gene clustering, dynamic shearing module identification, similar module clustering, and merging were sequentially performed. The relationship between gene modules and tuberculosis was assessed using gene significance (GS) values and module membership (MM) values to identify key modules. Gene modules highly correlated with the TB phenotype and differentially expressed and methylated genes in the TSS region were used to construct a protein-protein interaction (PPI) network. The PPI network was queried from the STRING online database, with interactions having a score >0.4 considered statistically significant (Szklarczyk et al., 2019). The resulting PPI network was visualized using Cytoscape (Shannon et al., 2003), and hub genes of the PPI network were identified using the maximum clique centrality (MCC) in the CytoHubba15 plugin (Chin et al., 2014).

### 2.4 Identification of potential methylated positions

Once candidate hub genes were determined, the corresponding differentially methylated positions were identified in the methylation microarray data. The Least Absolute Shrinkage and Selection Operator (LASSO) were then utilized to screen potential methylated positions, employing the “glmnet” package (Tibshirani, 1997). For differentially methylated regions (DMRs), we selected differentially methylated positions (DMPs) in the TSS region and used Support Vector Machine Recursive Feature Elimination (SVM-RFE) to screen potential methylated positions, implemented via the “e1071”package (Lin et al., 2012; Xia et al., 2021; Chen et al., 2022a). The predictor variable used in LASSO and SVM-RFE are the beta values from methylation microarray data, while the response variable corresponds to the phenotypic group information of the samples.

### 2.5 Construction of diagnostic classifier

All signature methylated positions selected are used to construct a classifier through various machine learning algorithms. All methods are performed through four-fold cross-validation and evaluated using ROC_AUC. The optimal hyperparameters are identified through grid search, and the efficacy of the classifier is tested using the external test set GSE145714. Data import and preprocessing are implemented using numpy and pandas in python 3.11.4. Machine learning algorithms were all implemented using sklearn, and the matplotlib library was used for result visualization.

### 2.6 Pyrosequencing

Pyrosequencing, a sequence analysis technology, was employed to quickly detect methylation frequency and qualitatively and quantitatively detect methylated positions in samples. In the investigation of pyrosequencing conducted in this study, verification was carried out using separate cohorts of 40 samples, including 22 TB and 18 HC samples. Primers were designed and evaluated using PyroMark Assay Design SW 2.0 (Qiagen) software. The samples of DNA were subjected to bisulfite conversion using the EZ DNA Methylation-Gold™ Kit (D5006, Zymo Research, California, United States). Subsequently, the PyroMark PCR Kit (Qiagen) was utilized for the polymerase chain reaction process, with a DNA input of 20 ng and a primer final concentration of 0.4 μM. The reaction involved three steps: initial denaturation at 98°C for 10 s, annealing at 55°C for 30 s, and extension at 72°C for 30 s, repeated for 35 cycles, followed by a final extension at 72°C for 1 min. Finally, the methylation level of the samples was analyzed on the PyroMark Q48 real-time quantitative pyrosequencing instrument (Qiagen). The plot was generated using R software (v.4.2.2) packages “ggpubr” (v0.4.0) and “ggplot2” (v3.4.2) through Hiplot Pro (https://hiplot.com.cn/), a comprehensive web service for biomedical data analysis and visualization (Li et al., 2022a). The pyrosequencing primers are listed in Supplementary Table S4.

### 2.7 Quantitative real-time methylation specific PCR (qMSP)

DNA samples were subjected to sulfite transformation using the EZ DNA Methylation Gold Kit (ZYMO RESEARCH, D5006, Los Angeles, CA, United States), following the manufacturer’s guidelines. The DNA concentration was measured using a spectrophotometer, and samples were stored at −20°C. The qMSP experiment involved 99 samples, including 49 TB and 50 HC samples, with an input of around 10 ng per sample. Primers and probes were designed based on the specific sequence of signature methylated positions, with ACTB serving as the reference gene. A 20 μL reaction system was prepared using Taq Pro HS Master Mix (Vazyme, Nanjing, China), and each sample was placed in triplicate wells. In this system, the final concentration of the primer is 0.2 μm, while the final concentration of the probe is 0.1 μm. The QuantStudio 7 Flex Real-Time PCR System (Applied Biosystems, Foster City, CA, United States) was used for sample amplification, employing a two-step PCR amplification program: predenaturation at 95°C for 30 s; 95°C for 10 s, 60°C for 30 s, repeated for 45 cycles. The specifics of the primers and probes are available in Supplementary Table S4. The details of the standard plasmid can be found in Supplementary Table S5.

### 2.8 Statistic analysis

The ΔCt value was utilized to determine the methylation level of candidate methylated positions in tissue samples. This value represents the normalized difference between the Ct value of the target position and the reference gene (ACTB) and the amount of DNA in the whole blood sample, i.e., ΔCt = Ct (target)–Ct (ACTB). Ct (target) and Ct (ACTB) are the average Ct values of three replicate wells in the qMSP results. A higher ΔCt value indicates a lower methylation level at the target position. The Ct value of the reference gene ACTB was used to verify the sample’s quality. If the Ct value of ACTB in the well exceeded 35, the sample was considered invalid. Differential analysis, AUC, specificity, and sensitivity calculations were all performed using SPSS 29.

## 3 Results

**FIGURE 1:**
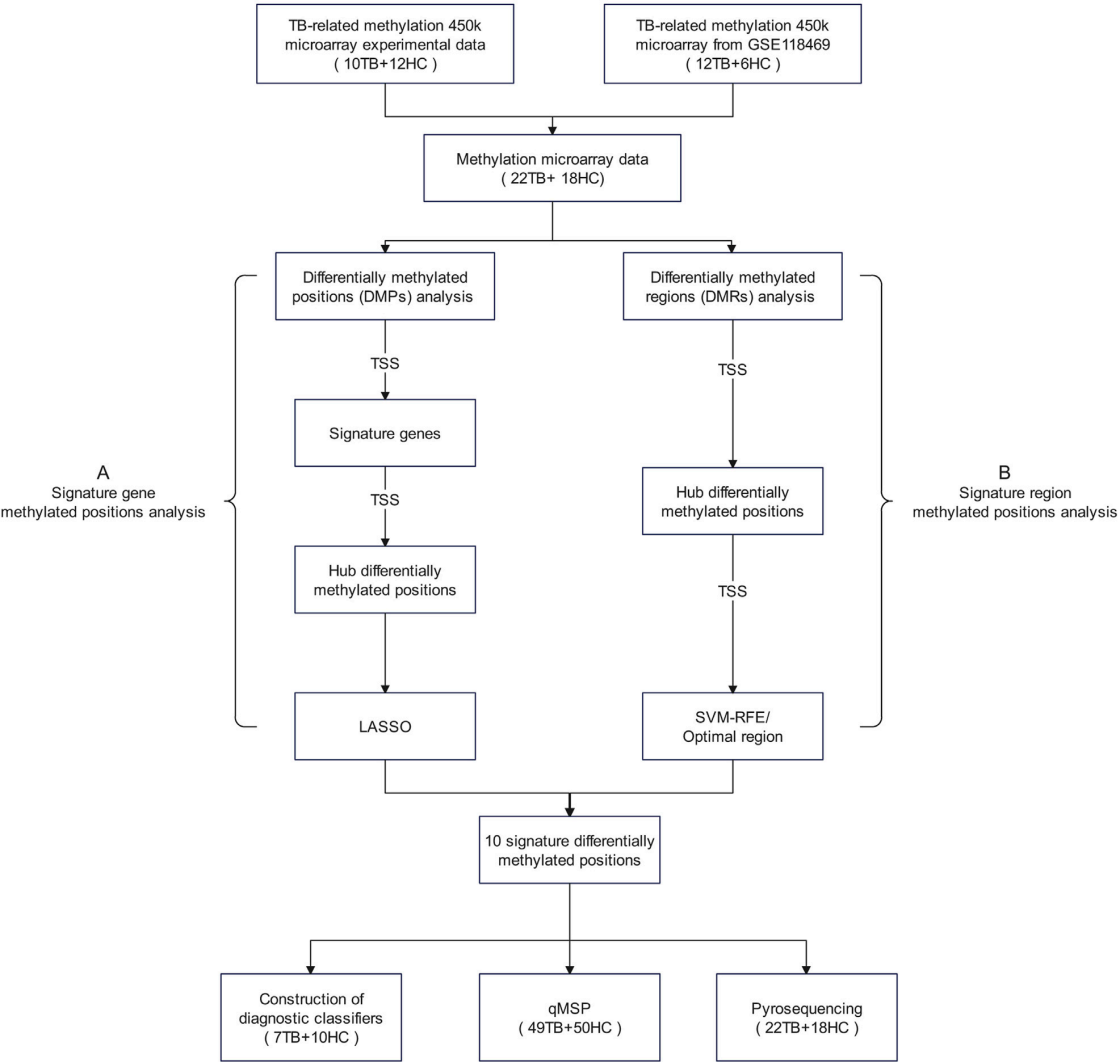
Research flowchart The term “signature methylated positions” in this study refers to a group of potential methylated positions identified through a stepwise screening process based on methylation microarray data. These methylated positions are considered to be closely associated with the phenotypes of tuberculosis, representing characteristic methylation patterns of tuberculosis. Abbreviations: DMPs, differentially methylated positions; DMRs, differentially methylated regions; LASSO, Least Absolute Shrinkage and Selection Operator; SVM-RFE, Support Vector Machine—Recursive Feature Elimination.

We amalgamated our own measured methylation microarray data with GSE118469 methylation microarray data from the Gene Expression Omnibus (GEO) database, totaling 40 blood samples’ methylation data, including 22 TB samples and 18 HC samples. The specific details of all datasets utilized in this article are available in Supplementary Table S1. To comprehensively investigate potential methylated positions, we employed two selection processes. Given that the TSS region constitutes the central segment of the promoter, our study concentrated on methylated positions situated in the TSS region. The flowchart of the research plan is shown in Figure 1.

### 3.1 Analysis process of potential gene methylated positions (process A)

**FIGURE 2:**
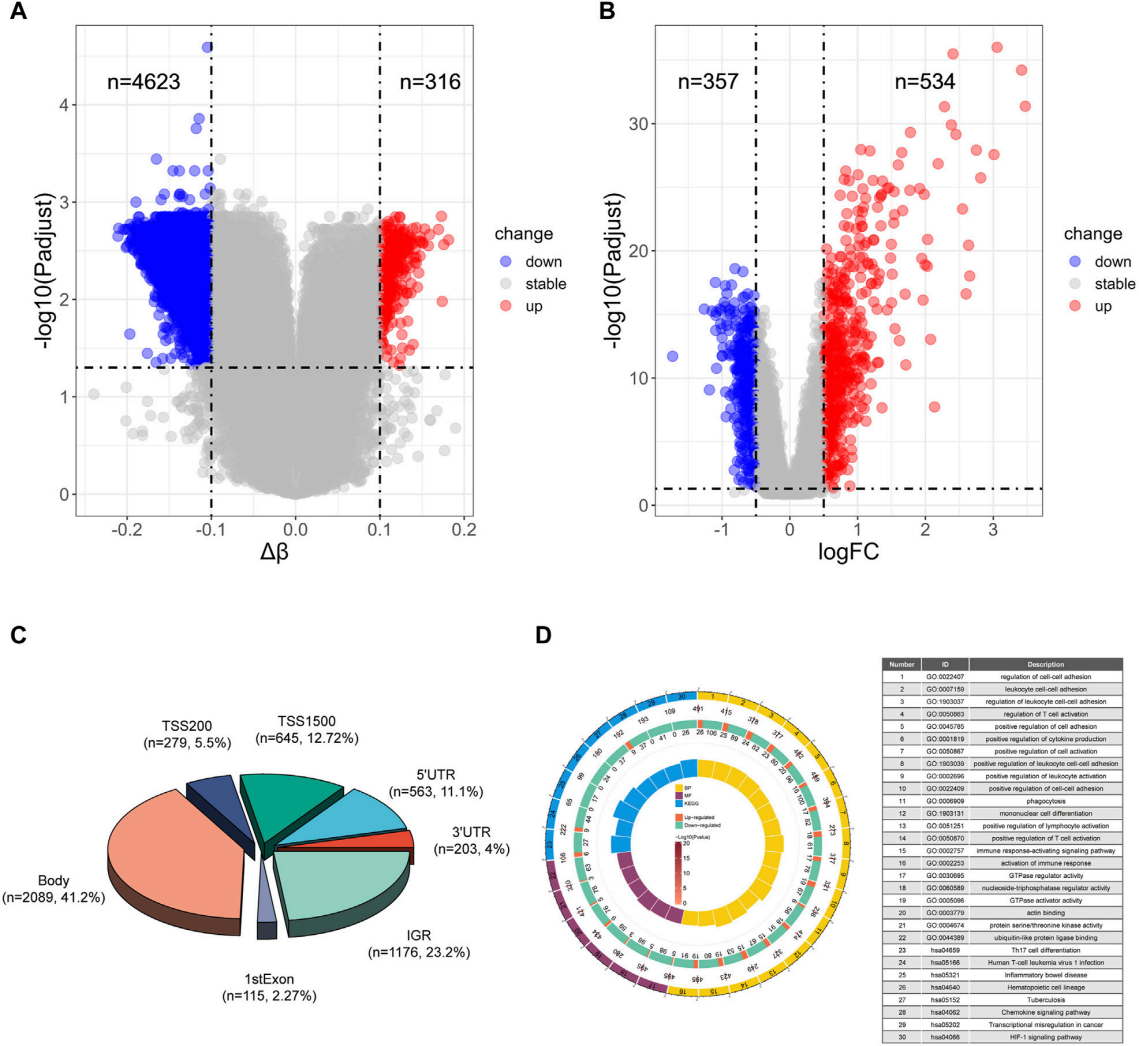
Differential analysis and enrichment analysis of TB and HC **(A)** Volcano plot of differentially methylated positions. **(B)** Volcano plot of differentially expressed genes. **(C)** Pie chart illustrating the regions of differentially methylated positions. **(D)** Top 16 pathways enriched in Biological Processes (BP), top 6 pathways enriched in Molecular Functions (MF), and top 8 pathways enriched in Kyoto Encyclopedia of Genes and Genomes (KEGG). The circles represent the serial number of the enrichment pathway, the enrichment *p*-value, the total number of genes in the pathway, the number of upregulated and downregulated genes, and the enrichment factor size.

To identify potential methylated positions on tuberculosis-associated genes, we executed process A. The outcomes reveal that the analysis of differentially methylated positions (DMPs) identified a total of 4939 DMPs, comprising 4623 hypomethylated positions and 316 hypermethylated positions (Figure 2A). This indicates that tuberculosis is primarily characterized by hypomethylation. Among the identified DMPs, 894 are located in the transcription start site (TSS) region, corresponding to 651 differentially methylated genes (DMGs). Subsequently, differentially gene expression analysis using data from the GEO database GSE83456 unveiled 891 differentially expressed genes (DEGs), with 357 genes exhibiting low expression and 534 genes exhibiting high expression (Figure 2B), suggesting that tuberculosis is primarily characterized by high gene expression.

Moreover, we illustrated the distribution of differential methylated positions in the gene region (Figure 2C). The results of the enrichment analysis for biological processes (BP) indicate that the genes containing DMPs are predominantly involved in immune-related biological processes, particularly enriched in pathways such as cell-cell adhesion, positive regulation of cytokine production, and positive regulation of T cell activation. Molecular function (MF) enrichment analysis reveals that DMPs in tuberculosis are mainly enriched in energy metabolism pathways like GTPase regulator activity and nucleoside-triphosphatase regulator activity. Notably, among the top 8 pathways, there is also enrichment of the ubiquitin-like protein ligase binding pathway, which plays a significant role in tuberculosis. Kyoto Encyclopedia of Genes and Genomes (KEGG) enrichment analysis shows that DMPs in tuberculosis are primarily enriched in pathways such as Th17 cell differentiation, Tuberculosis and Chemokine signaling pathway (Figure 2D).

**FIGURE 3:**
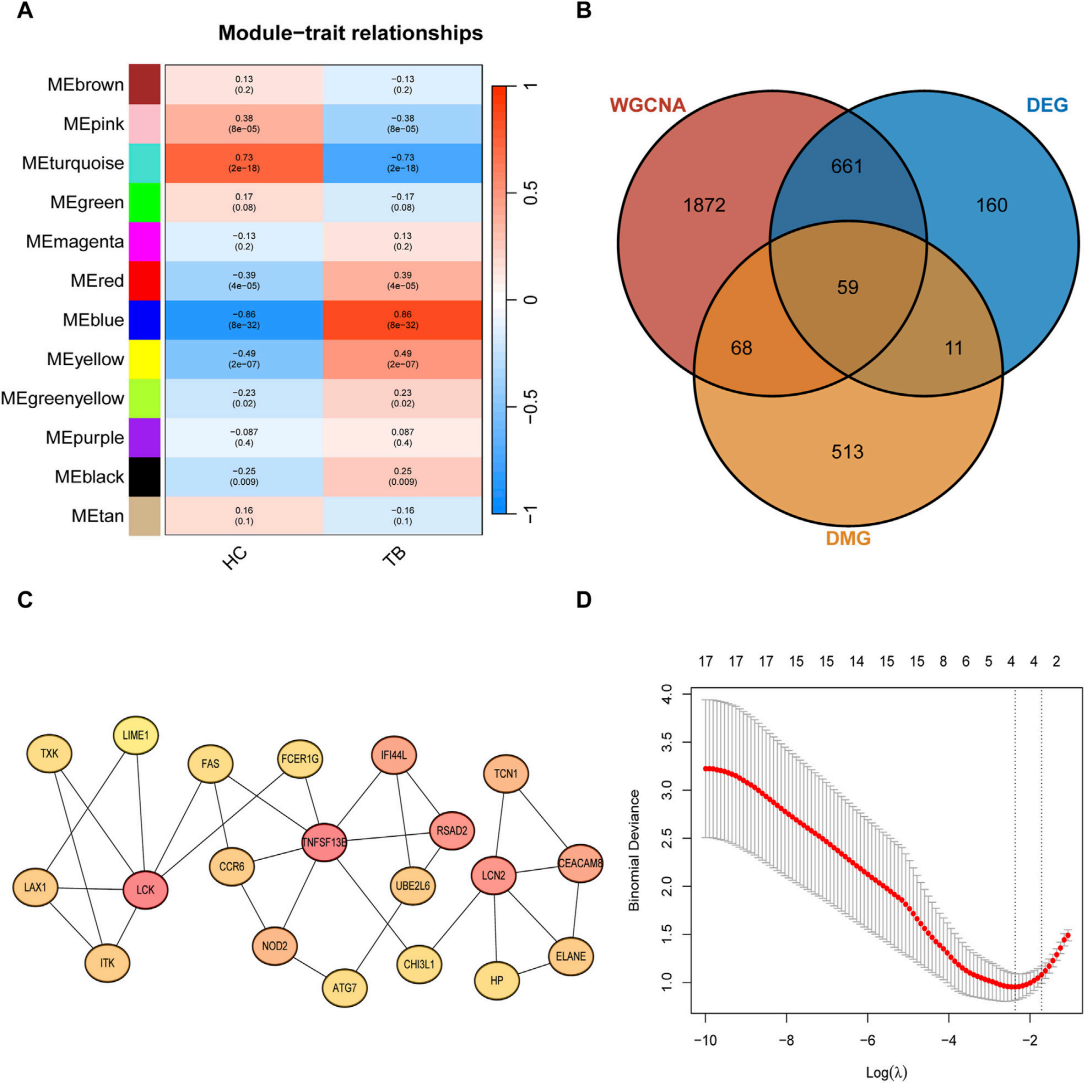
Signature gene methylated positions analysis **(A)** Clustered modules of WGCNA. **(B)** Venn plot showing the interaction between key module genes, differentially expressed genes (DEGs), and differentially methylated genes (DMGs) in the Transcription Start Site (TSS) region. **(C)** Top 20 feature genes in maximum clique centrality (MCC). **(D)** LASSO regularization path diagram, depicting the fitting effect of the model corresponding to different values of the regularization parameter (λ).

Through Weighted Correlation Network Analysis (WGCNA), all gene expression data were organized into 12 modules, and the correlation of each module with the TB phenotype was calculated (Figure 3A). The results revealed that MEblue (P = 8e-32, cor = 0.86) and MEturquoise (P = 2e-18, cor = −0.73) exhibited the highest correlation with TB, signifying them as key modules associated with TB. The detailed information of WGCNA can be found in Supplementary Figure S1. The MEblue module comprises 840 genes, and the MEturquoise module comprises 1,820 genes, summing up to 2,660 genes in these two modules. The intersection of key module genes, DEGs, and DMGs in the TSS region resulted in 59 intersecting genes (Figure 3B). Through Protein-Protein Interaction (PPI) analysis, the top 20 feature genes were selected (Figure 3C), corresponding to 23 key methylated positions in the TSS region. Utilizing the machine learning algorithm Lasso for variable selection, we identified 4 potential methylated positions: cg23213327 (*RSAD2*), cg17984638 (*TXK*), cg11554335(*UBE2L6*), cg07839457(*NLRC5*) (Figure 3D).

### 3.2 Analysis process of signature region methylated positions (process B)

**FIGURE 4:**
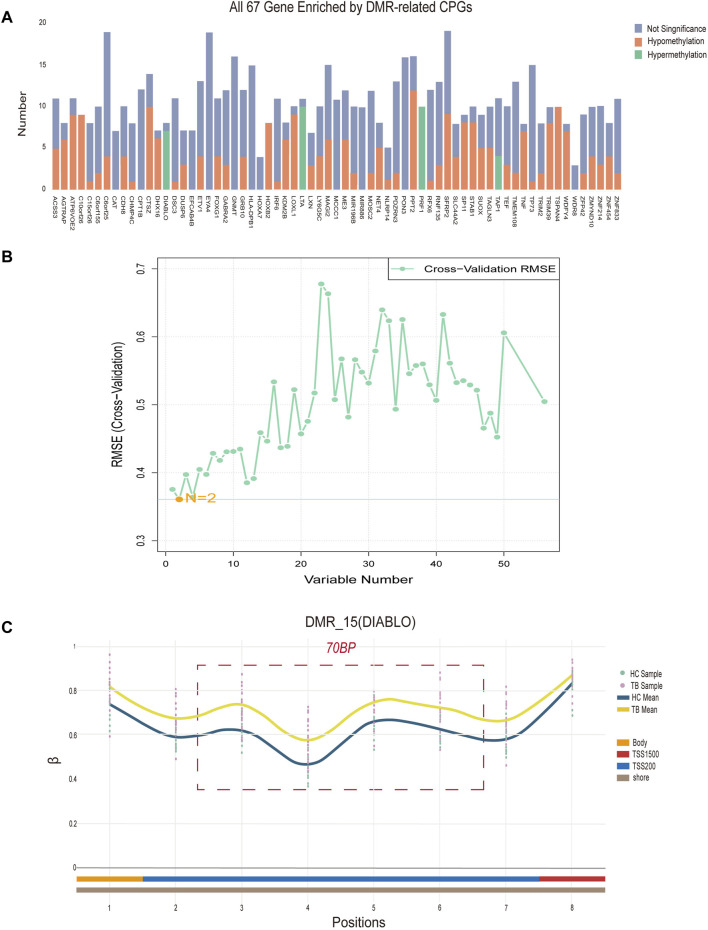
Signature region methylated positions analysis **(A)** Differentially methylated regions (DMRs) located on specific genes. “Number” denotes the quantity of methylated positions, “Not Significant” indicates the absence of a significant difference at the signature, “Hypomethylation” signifies notably decreased methylation at the signature, while “Hypermethylation” signifies notably increased methylation at the methylation. **(B)** The term “Variable Number” indicates the count of preserved methylated positions in the model, while RMSE (Cross-Validation) represents the Root Mean Square Error derived from cross-validation. **(C)** Data concerning all methylated positions within the optimal differentially methylated region, identifying positions 3, 4, 5, and 6 as the four potential methylated positions. The “TB Sample” and “HC Sample” denote the β values associated with each sample point in the methylation microarray data for tuberculosis (TB) and healthy control (HC) groups. “TB Mean” and “HC Mean” indicate the average β value for each methylation position in the TB and HC groups, respectively. The regions labeled “Body,” “TSS200,” and “TSS1500”correspond to the methylation positions within the *DIABLO* gene. Additionally, “Shore” designates the location of differentially methylated regions on CpG islands.

To identify potential methylated positions on tuberculosis-associated methylated regions, we implemented process B. The analysis of differentially methylated regions revealed a total of 69 DMRs, with 67 located on specific genes (Figure 4A), and the remaining two situated in intergenic regions (IGR). Among the DMRs, there are a total of 472 methylated positions in the TSS region. By intersecting the methylated positions in the TSS region selected from the DMRs with the differentially methylated positions (DMPs), we ultimately obtained 59 hub methylated positions (Figure 4B). Utilizing the Support Vector Machine-Recursive Feature Elimination (SVM-RFE) machine learning algorithm for variable selection, we determined that the model exhibited the smallest Root Mean Squared Error (RMSE) when only 2 methylated positions remained. These 2 positions, cg04552852 (*TSPAN4*) and cg09313705 (*HOXB2*), are considered as potential methylated positions and are located on two different DMRs.

Additionally, based on the density of differentially methylated positions, we identified the optimal differentially methylated region, containing the most 4 DMPs within 70bp (cg21805118, cg11804414, cg19529732, cg14094409). The 4 methylated positions in this region are considered as potential methylated positions and are situated on the *DIABLO* gene locus (Figure 4C). In conclusion, a total of 6 potential methylated positions were obtained from 3 DMRs through process B.

### 3.3 Construction of diagnostic classifier based on signature methylated positions

**FIGURE 5:**
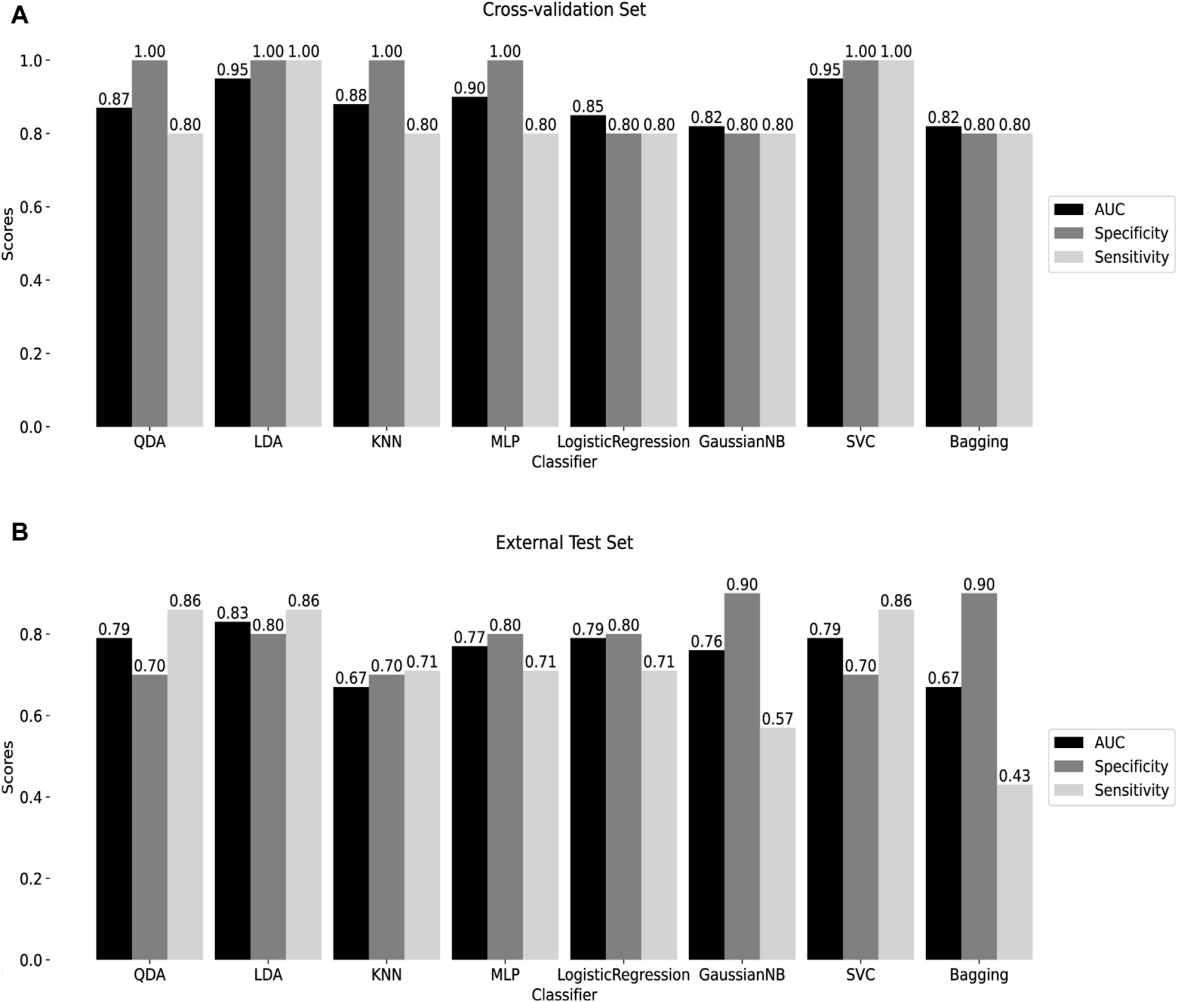
Construction of machine learning diagnostic classifier **(A)** Cross-validation results of 8 machine learning diagnostic classifiers. **(B)** Test results of the external test set GSE145714. QDA: Quadratic discriminant analysis. LDA: linear discriminant analysis. KNN: K-Nearest Neighbor. MLP: Multilayer perceptron. GaussianNB: Gaussian Naive Bayes. SVC: Support Vector Classification. Bagging: Bootstrap aggregating.

To assess the classification efficiency of the 10 signature methylated positions derived from two screening processes, a machine learning classifier was constructed. Detailed information about these positions is provided in Supplementary Table S2. Eight machine learning algorithms were applied for diagnostic classifier construction, and optimal model hyperparameters were determined through cross-validation and grid search, as outlined in Supplementary Table S3. Receiver Operating Characteristic (ROC) curves were utilized to assess diagnostic efficiency, and the areas under the ROC curves, along with specificity and sensitivity scores, were calculated. Cross-validation results demonstrated that the AUC of all classifiers exceeded 0.8, with both sensitivity and specificity surpassing 0.8 (Figure 5A), indicating the model’s high diagnostic value.

The performance of the classifiers was further evaluated using an external test set, GSE145714. Results indicated that, except for the KNN and Bagging classifiers, the AUC of the remaining classifiers surpassed 0.7, with both specificity and sensitivity exceeding 0.7, except for the GaussianNB and Bagging classifiers (Figure 5B). Notably, the LDA classifier exhibited robust diagnostic capabilities in both the training and external test sets, with AUC = 0.95, specificity = 1, and sensitivity = 1 in cross-validation; and AUC = 0.83, specificity = 0.80, and sensitivity = 0.86 in the external test set. Following this, the SVC, utilizing the radial basis kernel (rbf) function, demonstrated AUC = 0.95, specificity = 1, and sensitivity = 1 in cross-validation; and AUC = 0.79, specificity = 0.70, and sensitivity = 0.86 in the external test set. These findings suggest that the classifier, built on the 10 signature methylated positions, provides effective diagnostic classification, holding promise as a clinical tool for tuberculosis diagnosis.

### 3.4 Pyrosequencing validates signature methylated positions

We employed an additional 40 clinical samples (22TB and 18HC) to validate the screening outcomes through pyrosequencing and evaluated the methylation levels at signature methylated positions in whole blood. In this part, pyrosequencing confirmed 11 methylated positions, with 8 of them being among the previously screened 10 signature methylated positions (two of which were excluded from pyrosequencing due to the failure to design specific primers). The length of pyrosequencing being approximately 60 base pairs resulted in the detection of 3 additional methylated positions. These positions could potentially serve as methylation biomarkers for tuberculosis.

**FIGURE 6:**
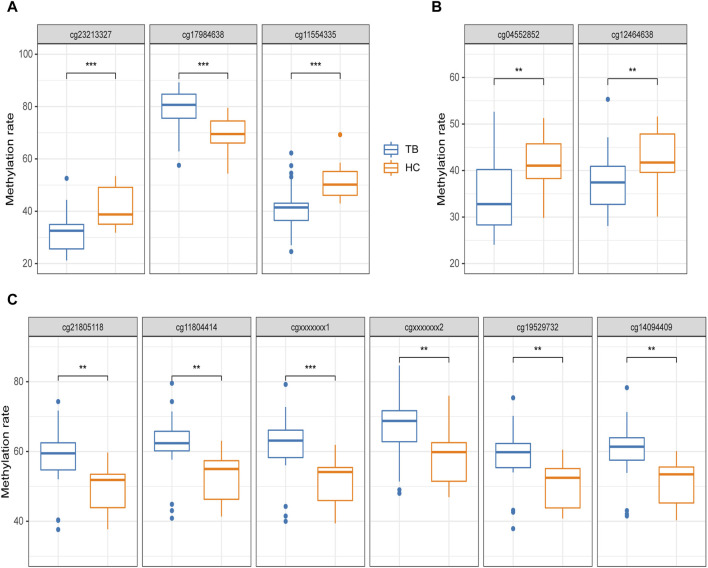
Pyrosequencing results **(A)** potential methylated positions obtained from the analysis of signature gene methylated positions. **(B)** Potential methylated positions obtained from the analysis of signature region methylated positions. **(C)** Potential methylated positions of the optimal differentially methylated region; cgxxxxxxx1 and cgxxxxxxx2 represent positions not designed in the methylation microarray. The differential analysis of Pyrosequencing results was uniformly conducted using t-tests. *p < 0.05, **p < 0.01, ***p < 0.001.

The pyrosequencing results indicated that, after analyzing four potential gene methylated positions (process A), 3 out of 4 exhibited significant differences in both TB and HC groups, excluding cg07839457 (*NLRC5*) was unable to have effective specific primers designed. Notably, cg17984638 (*TXK*) displayed significantly higher methylation, with an average methylation rate of 78.57% ± 8.5% for TB and 68.64% ± 7.38% for HC, while cg23213327 (*RSAD2*) and cg11554335 (*UBE2L6*) demonstrated significantly lower methylation, with average rates of 31.45% ± 8.27% and 41.6% ± 9.57% for TB, and 41.25% ± 7.72% and 51.17% ± 6.77% for HC, respectively. Furthermore, the observed trends in these three positions aligned with the methylation microarray data (Figure 6A).

Based on the analysis process of signature region methylated positions (Process B), 2 distinct potential methylated positions were identified (cg04552852 and cg09313705). cg04552852 (*TSPAN4*) exhibited significant differences in both the TB and HC groups, but cg09313705 (*HOXB2*) was unable to have effective specific primers designed. Furthermore, upon detecting cg04552852 (*TSPAN4*), the 9-bp spaced cg12464638 (*TSPAN4*) is simultaneously identified, both of which are located within the TSS region of *TSPAN4*. Both of these positions showed significantly lower methylation in the TB group, with average rates of 34.52% ± 7.65% and 37.37% ± 6.74% for TB, and 41.57% ± 5.33% and 42.94% ± 5.90% for HC, respectively (Figure 6B).

Furthermore, 2 additional methylated positions were detected in addition to the four positions identified in the optimal DMR. These 2 positions, not designed on the methylation microarray, demonstrated significantly high methylation in the TB group, consistent with the other four positions. The average methylation level of these 6 methylated positions in the TB group was 8.73% ± 0.45% higher than in the HC group (Figure 6C).

### 3.5 Verification of potential methylated positions by qMSP

**FIGURE 7:**
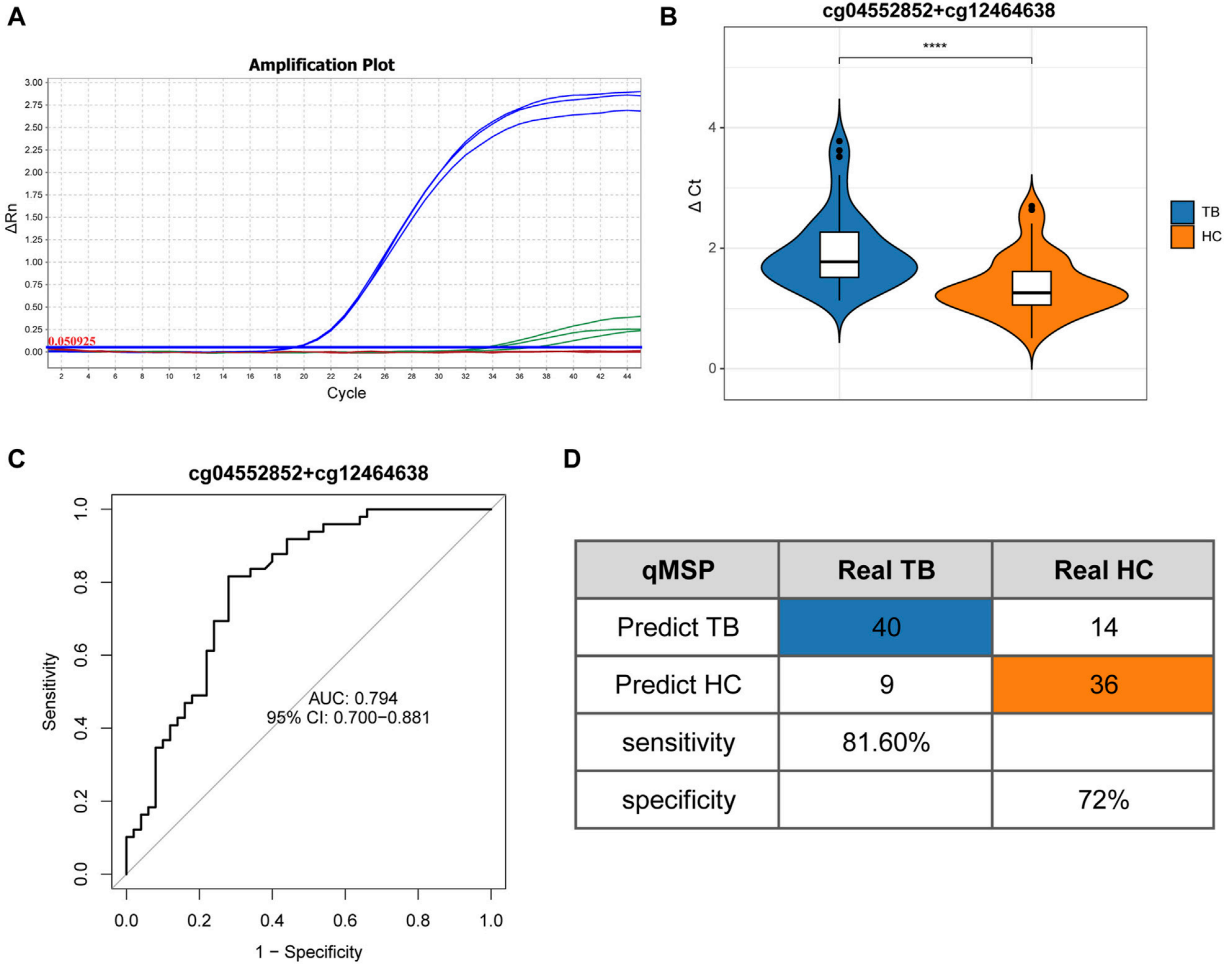
Quantitative real-time methylation specific PCR results **(A)** Amplification curve illustrates the qMSP results on triplicated samples. Blue lines represent the amplification of methylated plasmid templates, while green lines represent the absence of the amplification of unmethylated plasmid templates. **(B)** Differential analysis of ΔCt results between TB and HC. **(C)** ROC curve and AUC of TB and HC. **(D)** Confusion matrix, specificity, and sensitivity of TB and HC. Differential analysis of ΔCt results between TB and HC based on Mann-Whitney U test. *p < 0.05, **p < 0.01, ***p < 0.001, ****p < 0.0001.

To validate the screening results and ascertain the methylation levels of distinctive methylated positions in whole blood, we employed qMSP to detect the methylation of cg04552852 (*TSPAN4*) and cg12464638 (*TSPAN4*) in 49 cases of TB and 50 cases of HC. Table 1 lists the main information of sample. To verify the specificity of methylation detection, a test was carried out using the methylation probe. Synthetic methylated and unmethylated plasmids served as templates for the amplification process. The results indicated successful amplification of the methylated plasmid template and a lack of amplification for the unmethylated plasmid template. As a result, the methylation probe demonstrates high specificity (Figure 7A). The ΔCt value was used to indicate the methylation level at the position, with lower ΔCt values indicating higher methylation levels. The results demonstrated that the methylation level of cg04552852 and cg12464638 from *TSPAN4* in TB was significantly lower than that in HC (*p* < 0.0001) (Figure 7B). This finding aligns with the results of methylation microarray and pyrosequencing. Furthermore, the area under the ROC curve (AUC) was 0.794 (95%CI 0.700-0.881), with a sensitivity of 81.6% and a specificity of 72% (Figures 7C, D). The positive predictive value (PPV) is 74.07%, the negative predictive value (NPV) is 80%, and the accuracy is 76.77%.

**TABLE 1:** Clinical sample details.

Group	Characteristics	TB	HC
Methylation microarray data	Number	10	12
	Gender		
	Male	6	6
	Female	4	6
	Age (year)		
	<40	4	6
	40–60	6	6
	>60	0	0
	Diagnostic results		
	Pathogen diagnosis/Immunological diagnosis	Positive	Negative
Pyrosequencing	Number	22	18
	Gender		
	Male	15	12
	Female	7	6
	Age (year)		
	<40	6	7
	40–60	6	5
	>60	10	6
	Diagnostic results		
	Pathogen diagnosis/Immunological diagnosis	Positive	Negative
qMSP	Number	49	50
	Gender		
	Male	26	34
	Female	23	16
	Age (year)		
	<40	15	13
	40–60	15	16
	>60	19	21
	Pathogen diagnosis/Immunological diagnosis	Positive	Negative

## 4 Discussion

Tuberculosis is a long-lasting infectious ailment due to Mtb infection, posing a significant threat to human health. The number of identified tuberculosis cases falls notably short of the estimated patient count (incident cases), underscoring the limitations of current diagnostic approaches. Methylation, an emerging diagnostic technique, is increasingly gaining traction in cancer early detection. Various studies have devised methylation biomarkers for diverse cancers like lung, colorectal, cervical, and bladder cancers, which find application in clinical diagnosis (Han et al., 2019; Tang et al., 2019; Roy and Tiirikainen, 2020; Chang et al., 2023), such as the three-gene methylation biomarker (*SHOX2/RASSF1A/PTGER4*) for lung cancer (Kneip et al., 2011; Weiss et al., 2017; Malpeli et al., 2019). Although methylation’s potential as a biomarker exists in infectious diseases, no applicable diagnostic method for clinical use has been established. Given tuberculosis’s nature as a chronic immune ailment, sharing similarities with lung cancer in the immune microenvironment (Ramakrishnan, 2012; Mayer-Barber and Barber, 2015; Cohen et al., 2022), methylation likely holds promise for tuberculosis diagnosis. The diagnostic method qMSP, employed in clinical, can potentially be adapted for tuberculosis diagnosis. Accordingly, this study utilized methylation microarray data for two screening processes: signature gene methylated position analysis and signature region methylated position analysis, leading to the identification of 10 signature methylated positions. Multiple machine learning algorithms were employed to formulate a tuberculosis diagnostic classifier, subsequently validated for diagnostic efficiency using the external test set GSE145714. The results indicate commendable specificity and sensitivity in the diagnostic classifier, presenting it as a potential clinical diagnostic tool. Furthermore, pyrosequencing validated the 10 signature methylated positions, detecting 8 successfully and uncovering 3 additional positions. All 11 positions exhibited significant differences between TB and healthy control (HC) groups, consistent with methylation microarray results, affirming their potential as tuberculosis diagnostic biomarkers. Lastly, qMSP detected methylation of cg04552852 and cg12464638 from *TSPAN4* in 99 whole blood samples, with an AUC of 0.794, specificity of 0.720, and sensitivity of 0.816, illustrating the efficacy of assessing the methylation status of cg04552852 and cg12464638 in whole blood for tuberculosis diagnosis.

Methylation diagnostic markers, an evolving diagnostic approach, offer distinct advantages in disease detection: 1) Early detection is feasible as methylation modifications precede gene expression; 2) DNA methylation alterations demonstrate relative stability; 3) The examination is safe, non-invasive, and devoid of trauma or side effects; 4) The procedure is straightforward, typically involving peripheral blood extraction without the need for special preparation (Ziegler et al., 2012; Yu et al., 2022).

DNA methylation modifications in promoter regions play a role in regulating downstream gene expression, demonstrating a negative correlation (Klose and Bird, 2006; Héberlé and Bardet, 2019; Isbel et al., 2022). Among the pyrosequencing-validated positions in this study, excluding the four potential methylated positions in the optimal differentially methylated region, all others adhere to this characteristic, for further details, please refer to the Supplementary Figures S2–S4. Notably, cg11554335, situated on the *UBE2L6* gene, exhibits low methylation in tuberculosis (TB) and elevated gene expression. *UBE2L6*, an E2 ubiquitin-conjugating enzyme, has been identified as a biomarker for active tuberculosis, displaying heightened expression in the whole blood of tuberculosis patients (Gao et al., 2021). *UBE2L6* can also influence the tuberculosis immune response by post-translationally modifying phagosome-associated proteins through ubiquitination (Zhang et al., 2023), suggesting potential regulation by methylation. Enrichment analysis results reveal that differentially methylated positions in this gene are enriched in the ubiquitin-like protein ligase binding pathway, with 63 genes in this pathway displaying significant hypomethylation. Recent studies indicate that tuberculosis proteins may exploit the host ubiquitination system to suppress immunity (Wang et al., 2015; Wang et al., 2020; Chai et al., 2022). Consequently, the ubiquitination modification pathway in the human body may be regulated by methylation, potentially impacting tuberculosis development. We are presently enhancing the design of the probe and the PCR methodology, specifically customized for this target location, anticipating successful detection in the immediate future.

The 2 distinctive methylated positions, cg04552852 and cg12464638, reside on the *TSPAN4* gene, displaying reduced methylation and heightened expression in TB. This suggests that the *TSPAN4* gene may undergo regulation via methylation modifications. Recent studies link the *TSPAN4* gene to the formation of migrasomes, known to recruit monocytes and stimulate angiogenesis (Zhang et al., 2022). Given the relevance of angiogenesis to tuberculosis, where anti-angiogenic drugs effectively reduce bacterial burden in granulomas (Oehlers et al., 2015), *TSPAN4* may play a role in tuberculosis development.

Current investigations into tuberculosis methylation primarily focus on regulatory mechanisms. Several studies reveal that Mtb induces host methylation modifications through methyltransferases, enabling evasion of the host’s immune system (Sharma et al., 2015; Yaseen et al., 2015; Sharma et al., 2016). However, limited efforts have been made in advancing diagnostic markers for tuberculosis. Research suggests that abnormal methylation in the promoter regions of TLR2 or genes related to vitamin D metabolism in peripheral blood is associated with tuberculosis risk (Chen et al., 2014; Wang et al., 2018; Chen et al., 2022b). Furthermore, two differentially methylated regions (DMRs), chr3: 195635643-195636243 and chr6: 29691631-29692475, exhibit an AUC of 0.838, sensitivity of 0.645, and specificity of 0.903 in TB and HC (Lyu et al., 2022). Existing studies either lack methylation microarray analysis or focus on specific cells. Moreover, current studies employ Next-Generation Sequencing for validation, a process entailing significant expense and complexity, posing constraints in clinical applications. Consequently, we have, for the first time, utilized qMSP technology in tuberculosis diagnosis. The gold standard for tuberculosis diagnosis is pathogen diagnosis (including Xpert MTB/RIF and AFB), with qMSP testing showing high consistency with it. The diagnostic agreement rates with Xpert MTB/RIF and AFB are 95% and 95.2%, respectively. Methylated detection offers a shorter processing time compared to pathogen diagnostic methods and eliminates the need for special preparation before testing. It does not require fresh whole blood or overnight culture compared to immunological diagnosis. Cost estimation suggests that methylated detection is more cost-effective than pathogen and immunological testing, requiring only an RT-PCR instrument for quantification without the need for advanced laboratory facilities or specialized equipment. This could be significant for tuberculosis diagnosis in economically disadvantaged areas. Furthermore, methylated detection may be effective in detecting tuberculosis patients with co-infections such as HIV/AIDS and those with autoimmune diseases, although further exploration is needed. These findings indicate its considerable potential in clinical diagnosis.

In this study, we effectively identified methylation differences in cg04552852 and cg12464638 from *TSPAN4* using qMSP. However, focusing solely on the detection of two methylated positions may limit the accuracy of the diagnosis. To overcome this limitation, we plan to optimize the methods and probes to effectively validate the screened methylation positions. The validation of individual methylated positions presents a challenge due to the absence of other methylation positions within a 30bp proximity, making it difficult to ensure probe specificity. To overcome these challenges, it is essential to optimize probe design and PCR methods to achieve precise targeting of individual methylation positions. Nonetheless, a study has demonstrated an effective qMSP method for single methylated position specificity (Yu et al., 2019), and employing Locked Nucleic Acid (LNA) modifications is also a viable approach to enhance specificity (Petersen and Wengel, 2003). Thus, combining these methods to design positions recognizing single methylated position and using a multiplex qMSP system with multiple positions can enhance the accuracy of tuberculosis methylation marker diagnosis. In addition, in this study, the sample numbers for external test sets and clinical validation remain limited, which may impact the generalizability and statistical accuracy of this method. Therefore, further recruitment is warranted to validate the specificity, sensitivity, and robustness of the experimental results. Next, we will also focus on optimizing the qMSP method and its reaction system, while investigating potential biases and the clinical applicability in various settings.

In summary, DNA methylation serves as a biomarker for tuberculosis diagnosis, with whole blood DNA methylation status detection proving effective. Additionally, methylation acts as a regulatory marker for immunopathology (Chen et al., 2020; Khadela et al., 2022), and it holds potential as a therapeutic and prognostic marker in various diseases (Gampenrieder et al., 2018; Guo et al., 2019; Chen et al., 2020; Liang et al., 2022), with the possibility of future application in tuberculosis. Optimization and application of methylation detection methods are beneficial for diagnosing tuberculosis in high-incidence and economically challenged regions. Furthermore, further exploration of methylation detection may aid in diagnosing tuberculosis co-infected patients, such as those with HIV or autoimmune diseases. In conclusion, methylation detection can facilitate early diagnosis, monitoring, and treatment of tuberculosis patients, finally meeting the requirements of ending TB strategy.

## 5 Conclusion

We have identified 10 signature methylated positions, from which a diagnostic classifier has been developed as a potential tool for clinical diagnosis. Furthermore, we have successfully validated 11 methylated positions using pyrosequencing, potentially serving as biomarkers for tuberculosis diagnosis. Importantly. We have introduced a novel method for detecting the *TSPAN4* TSS region (cg04552852 and cg12464638) in whole blood samples, offering an effective means for tuberculosis diagnosis.

## Data Availability

The datasets presented in this study can be found in online repositories. The names of the repository/repositories and accession number(s) can be found in the article/Supplementary Material.

## Abbreviations

TB, Tuberculosis; HC, Healthy Control; Mtb, *Mycobacterium tuberculosis*; qMSP, Quantitative real-time methylation specific PCR; GEO, Gene Expression Omnibus; DMPs, differentially methylated positions; DMGs, differentially methylated genes; DEGs, differentially expressed genes; DMRs, differentially methylated regions; WGCNA, Weighted correlation network analysis; CpG, Cytosine-phosphate-guanine; PPI, Protein-Protein Interaction Networks; Lasso, least absolute shrinkage and selection operator; SVM-RFE, Support Vector Machine—Recursive Feature Elimination; ROC, Receiver Operating Characteristic; AUC, Area Under Curve.

## References

[B1] Baylin S. B. Jones P. A. (2011). A decade of exploring the cancer epigenome - biological and translational implications. Nat. Rev. Cancer 11, 726–734. 10.1038/nrc3130 21941284 PMC3307543

[B2] Capper D. Jones D. T. W. Sill M. Hovestadt V. Schrimpf D. Sturm D. (2018). DNA methylation-based classification of central nervous system tumours. Nature 555, 469–474. 10.1038/nature26000 29539639 PMC6093218

[B3] Chai Q. Yu S. Zhong Y. Lu Z. Qiu C. Yu Y. (2022). A bacterial phospholipid phosphatase inhibits host pyroptosis by hijacking ubiquitin. Science 378, eabq0132. 10.1126/science.abq0132 36227980

[B4] Chang L. Wang D. Han Y. Diao Z. Chen Y. Li J. (2023). External quality assessment for detection of colorectal cancer by Septin9 DNA methylation in clinical laboratories. Clin. Chim. Acta 552, 117663. 10.1016/j.cca.2023.117663 38008152

[B5] Chen Y. C. Hsiao C. C. Chen C. J. Chao T. Y. Leung S. Y. Liu S. F. (2014). Aberrant Toll-like receptor 2 promoter methylation in blood cells from patients with pulmonary tuberculosis. J. Infect. 69, 546–557. 10.1016/j.jinf.2014.08.014 25218055

[B6] Chen Y. C. Hsiao C. C. Chen T. W. Wu C. C. Chao T. Y. Leung S. Y. (2020). Whole genome DNA methylation analysis of active pulmonary tuberculosis disease identifies novel epigenotypes: PARP9/miR-505/RASGRP4/GNG12 gene methylation and clinical phenotypes. Int. J. Mol. Sci. 21, 3180. 10.3390/ijms21093180 32365959 PMC7246806

[B7] Chen H. Zhang J. Sun X. Wang Y. Qian Y. (2022a). Mitophagy-mediated molecular subtypes depict the hallmarks of the tumour metabolism and guide precision chemotherapy in pancreatic adenocarcinoma. Front. Cell Dev. Biol. 10, 901207. 10.3389/fcell.2022.901207 35938160 PMC9353335

[B8] Chen Y. Peng A. Z. Li K. Liu L. Zhang F. Chen J. (2022b). Association between promoter methylation of vitamin D metabolic pathway genes and tuberculosis and diabetes comorbidity in a Chinese han population: a case-control study. J. Inflamm. Res. 15, 6831–6842. 10.2147/jir.S393224 36583132 PMC9793733

[B9] Chin C. H. Chen S. H. Wu H. H. Ho C. W. Ko M. T. Lin C. Y. (2014). cytoHubba: identifying hub objects and sub-networks from complex interactome. BMC Syst. Biol. 8 (Suppl. 4), S11. 10.1186/1752-0509-8-s4-s11 25521941 PMC4290687

[B10] Cohen S. B. Gern B. H. Urdahl K. B. (2022). The tuberculous granuloma and preexisting immunity. Annu. Rev. Immunol. 40, 589–614. 10.1146/annurev-immunol-093019-125148 35130029

[B11] Dawson M. A. Kouzarides T. (2012). Cancer epigenetics: from mechanism to therapy. Cell 150, 12–27. 10.1016/j.cell.2012.06.013 22770212

[B12] Gampenrieder S. P. Rinnerthaler G. Hackl H. Pulverer W. Weinhaeusel A. Ilic S. (2018). DNA methylation signatures predicting bevacizumab efficacy in metastatic breast cancer. Theranostics 8, 2278–2288. 10.7150/thno.23544 29721079 PMC5928889

[B13] Gao J. Li C. Li W. Chen H. Fu Y. Yi Z. (2021). Increased UBE2L6 regulated by type 1 interferon as potential marker in TB. J. Cell Mol. Med. 25, 11232–11243. 10.1111/jcmm.17046 34773365 PMC8650027

[B14] Global tuberculosis report 2023 (2023). Geneva world health organization. Licence CC BY-NC-SA 3.0 IGO.

[B15] Guo J. Zhang X. Chen X. Cai Y. (2022). Proteomics in biomarker discovery for tuberculosis: current status and future perspectives. Front. Microbiol. 13, 845229. 10.3389/fmicb.2022.845229 35558124 PMC9087271

[B16] Guo W. Zhu L. Zhu R. Chen Q. Wang Q. Chen J. Q. (2019). A four-DNA methylation biomarker is a superior predictor of survival of patients with cutaneous melanoma. Elife 8, e44310. 10.7554/eLife.44310 31169496 PMC6553943

[B17] Gu Z. Eils R. Schlesner M. (2016). Complex heatmaps reveal patterns and correlations in multidimensional genomic data. Bioinformatics 32, 2847–2849. 10.1093/bioinformatics/btw313 27207943

[B18] Gu Z. Gu L. Eils R. Schlesner M. Brors B. (2014). Circlize Implements and enhances circular visualization in R. Bioinformatics 30, 2811–2812. 10.1093/bioinformatics/btu393 24930139

[B19] Han Y. D. Oh T. J. Chung T. H. Jang H. W. Kim Y. N. An S. (2019). Early detection of colorectal cancer based on presence of methylated syndecan-2 (SDC2) in stool DNA. Clin. Epigenetics 11, 51. 10.1186/s13148-019-0642-0 30876480 PMC6419806

[B20] Hao X. Luo H. Krawczyk M. Wei W. Wang W. Wang J. (2017). DNA methylation markers for diagnosis and prognosis of common cancers. Proc. Natl. Acad. Sci. U. S. A. 114, 7414–7419. 10.1073/pnas.1703577114 28652331 PMC5514741

[B21] HéBERLé É. Bardet A. F. (2019). Sensitivity of transcription factors to DNA methylation. Essays Biochem. 63, 727–741. 10.1042/ebc20190033 31755929 PMC6923324

[B22] Isbel L. Grand R. S. SchüBELER D. (2022). Generating specificity in genome regulation through transcription factor sensitivity to chromatin. Nat. Rev. Genet. 23, 728–740. 10.1038/s41576-022-00512-6 35831531

[B23] Khadela A. Chavda V. P. Postwala H. Shah Y. Mistry P. Apostolopoulos V. (2022). Epigenetics in tuberculosis: immunomodulation of host immune response. Vaccines (Basel) 10, 1740. 10.3390/vaccines10101740 36298605 PMC9611989

[B24] Klose R. J. Bird A. P. (2006). Genomic DNA methylation: the mark and its mediators. Trends Biochem. Sci. 31, 89–97. 10.1016/j.tibs.2005.12.008 16403636

[B25] Kneip C. Schmidt B. Seegebarth A. Weickmann S. Fleischhacker M. Liebenberg V. (2011). SHOX2 DNA methylation is a biomarker for the diagnosis of lung cancer in plasma. J. Thorac. Oncol. 6, 1632–1638. 10.1097/JTO.0b013e318220ef9a 21694641

[B26] Langfelder P. Horvath S. (2008). WGCNA: an R package for weighted correlation network analysis. BMC Bioinforma. 9, 559. 10.1186/1471-2105-9-559 PMC2631488 19114008

[B27] Liang L. Zhang Y. Li C. Liao Y. Wang G. Xu J. (2022). Plasma cfDNA methylation markers for the detection and prognosis of ovarian cancer. EBioMedicine 83, 104222. 10.1016/j.ebiom.2022.104222 35973389 PMC9396542

[B28] Li J. Miao B. Wang S. Dong W. Xu H. Si C. (2022a). Hiplot: a comprehensive and easy-to-use web service for boosting publication-ready biomedical data visualization. Brief. Bioinform 23, bbac261. 10.1093/bib/bbac261 35788820

[B29] Lin X. Yang F. Zhou L. Yin P. Kong H. Xing W. (2012). A support vector machine-recursive feature elimination feature selection method based on artificial contrast variables and mutual information. J. Chromatogr. B Anal. Technol. Biomed. Life Sci. 910, 149–155. 10.1016/j.jchromb.2012.05.020 22682888

[B30] Li Z. Mei Z. Ding S. Chen L. Li H. Feng K. (2022b). Identifying methylation signatures and rules for COVID-19 with machine learning methods. Front. Mol. Biosci. 9, 908080. 10.3389/fmolb.2022.908080 35620480 PMC9127386

[B31] Lyu M. Zhou J. Jiao L. Wang Y. Zhou Y. Lai H. (2022). Deciphering a TB-related DNA methylation biomarker and constructing a TB diagnostic classifier. Mol. Ther. Nucleic Acids 27, 37–49. 10.1016/j.omtn.2021.11.014 34938605 PMC8645423

[B32] Malpeli G. Innamorati G. Decimo I. Bencivenga M. Nwabo Kamdje A. H. Perris R. (2019). Methylation dynamics of RASSF1A and its impact on cancer. Cancers (Basel) 11, 959. 10.3390/cancers11070959 31323949 PMC6678546

[B33] Mayer-Barber K. D. Barber D. L. (2015). Innate and adaptive cellular immune responses to *Mycobacterium tuberculosis* infection. Cold Spring Harb. Perspect. Med. 5, a018424. 10.1101/cshperspect.a018424 26187873 PMC4665043

[B34] Mcnerney R. Maeurer M. Abubakar I. Marais B. Mchugh T. D. Ford N. (2012). Tuberculosis diagnostics and biomarkers: needs, challenges, recent advances, and opportunities. J. Infect. Dis. 205 (Suppl. 2), S147–S158. 10.1093/infdis/jir860 22496353

[B35] Morris T. J. Butcher L. M. Feber A. Teschendorff A. E. Chakravarthy A. R. Wojdacz T. K. (2014). ChAMP: 450k chip analysis methylation pipeline. Bioinformatics 30, 428–430. 10.1093/bioinformatics/btt684 24336642 PMC3904520

[B36] Nogueira B. M. F. Krishnan S. Barreto-Duarte B. AraúJO-Pereira M. Queiroz A. T. L. Ellner J. J. (2022). Diagnostic biomarkers for active tuberculosis: progress and challenges. EMBO Mol. Med. 14, e14088. 10.15252/emmm.202114088 36314872 PMC9728055

[B37] Oehlers S. H. Cronan M. R. Scott N. R. Thomas M. I. Okuda K. S. Walton E. M. (2015). Interception of host angiogenic signalling limits mycobacterial growth. Nature 517, 612–615. 10.1038/nature13967 25470057 PMC4312197

[B38] Papanicolau-Sengos A. Aldape K. (2022). DNA methylation profiling: an emerging paradigm for cancer diagnosis. Annu. Rev. Pathol. 17, 295–321. 10.1146/annurev-pathol-042220-022304 34736341

[B39] Petersen M. Wengel J. (2003). LNA: a versatile tool for therapeutics and genomics. Trends Biotechnol. 21, 74–81. 10.1016/s0167-7799(02)00038-0 12573856

[B40] Portela A. Esteller M. (2010). Epigenetic modifications and human disease. Nat. Biotechnol. 28, 1057–1068. 10.1038/nbt.1685 20944598

[B41] Ramakrishnan L. (2012). Revisiting the role of the granuloma in tuberculosis. Nat. Rev. Immunol. 12, 352–366. 10.1038/nri3211 22517424

[B42] Ritchie M. E. Phipson B. Wu D. Hu Y. Law C. W. Shi W. (2015). Limma powers differential expression analyses for RNA-sequencing and microarray studies. Nucleic Acids Res. 43, e47. 10.1093/nar/gkv007 25605792 PMC4402510

[B43] Roy D. Tiirikainen M. (2020). Diagnostic power of DNA methylation classifiers for early detection of cancer. Trends Cancer 6, 78–81. 10.1016/j.trecan.2019.12.006 32061307 PMC7188195

[B44] Shannon P. Markiel A. Ozier O. Baliga N. S. Wang J. T. Ramage D. (2003). Cytoscape: a software environment for integrated models of biomolecular interaction networks. Genome Res. 13, 2498–2504. 10.1101/gr.1239303 14597658 PMC403769

[B45] Sharma G. Sowpati D. T. Singh P. Khan M. Z. Ganji R. Upadhyay S. (2016). Genome-wide non-CpG methylation of the host genome during *M. tuberculosis* infection. Sci. Rep. 6, 25006. 10.1038/srep25006 27112593 PMC4845000

[B46] Sharma G. Upadhyay S. Srilalitha M. Nandicoori V. K. Khosla S. (2015). The interaction of mycobacterial protein Rv2966c with host chromatin is mediated through non-CpG methylation and histone H3/H4 binding. Nucleic Acids Res. 43, 3922–3937. 10.1093/nar/gkv261 25824946 PMC4417171

[B47] Szklarczyk D. Gable A. L. Lyon D. Junge A. Wyder S. Huerta-Cepas J. (2019). STRING v11: protein-protein association networks with increased coverage, supporting functional discovery in genome-wide experimental datasets. Nucleic Acids Res. 47, D607–D613. 10.1093/nar/gky1131 30476243 PMC6323986

[B48] Tang L. Liou Y. L. Wan Z. R. Tang J. Zhou Y. Zhuang W. (2019). Aberrant DNA methylation of PAX1, SOX1 and ZNF582 genes as potential biomarkers for esophageal squamous cell carcinoma. Biomed. Pharmacother. 120, 109488. 10.1016/j.biopha.2019.109488 31629253

[B49] Tian Y. Morris T. J. Webster A. P. Yang Z. Beck S. Feber A. (2017). ChAMP: updated methylation analysis pipeline for Illumina BeadChips. Bioinformatics 33, 3982–3984. 10.1093/bioinformatics/btx513 28961746 PMC5860089

[B50] Tibshirani R. (1997). The lasso method for variable selection in the Cox model. Stat. Med. 16, 385–395. 10.1002/(sici)1097-0258(19970228)16:4<385::aid-sim380>3.0.co;2-3 9044528

[B51] Wang J. Li B. X. Ge P. P. Li J. Wang Q. Gao G. F. (2015). *Mycobacterium tuberculosis* suppresses innate immunity by coopting the host ubiquitin system. Nat. Immunol. 16, 237–245. 10.1038/ni.3096 25642820

[B52] Wang L. Wu J. Li J. Yang H. Tang T. Liang H. (2020). Host-mediated ubiquitination of a mycobacterial protein suppresses immunity. Nature 577, 682–688. 10.1038/s41586-019-1915-7 31942069

[B53] Wang M. Kong W. He B. Li Z. Song H. Shi P. (2018). Vitamin D and the promoter methylation of its metabolic pathway genes in association with the risk and prognosis of tuberculosis. Clin. Epigenetics 10, 118. 10.1186/s13148-018-0552-6 30208925 PMC6136159

[B54] Weiss G. Schlegel A. Kottwitz D. KöNIG T. Tetzner R. (2017). Validation of the SHOX2/PTGER4 DNA methylation marker panel for plasma-based discrimination between patients with malignant and nonmalignant lung disease. J. Thorac. Oncol. 12, 77–84. 10.1016/j.jtho.2016.08.123 27544059 PMC5226366

[B55] Xia Y. Ying S. Jin R. Wu H. Shen Y. Yin T. (2021). Application of a classifier combining bronchial transcriptomics and chest computed tomography features facilitates the diagnostic evaluation of lung cancer in smokers and nonsmokers. Int. J. Cancer 149, 1290–1301. 10.1002/ijc.33675 33963762

[B56] Yaseen I. Kaur P. Nandicoori V. K. Khosla S. (2015). Mycobacteria modulate host epigenetic machinery by Rv1988 methylation of a non-tail arginine of histone H3. Nat. Commun. 6, 8922. 10.1038/ncomms9922 26568365

[B57] Yu H. Bai L. Tang G. Wang X. Huang M. Cao G. (2019). Novel assay for quantitative analysis of DNA methylation at single-base resolution. Clin. Chem. 65, 664–673. 10.1373/clinchem.2018.298570 30737203

[B58] Yu X. Cen L. Chen Y. A. Markowitz J. Shaw T. I. Tsai K. Y. (2022). Tumor expression quantitative trait methylation screening reveals distinct CpG panels for deconvolving cancer immune signatures. Cancer Res. 82, 1724–1735. 10.1158/0008-5472.Can-21-3113 35176128 PMC9064917

[B59] Zhang Q. A. Ma S. Li P. Xie J. (2023). The dynamics of *Mycobacterium tuberculosis* phagosome and the fate of infection. Cell Signal 108, 110715. 10.1016/j.cellsig.2023.110715 37192679

[B60] Zhang C. Li T. Yin S. Gao M. He H. Li Y. (2022). Monocytes deposit migrasomes to promote embryonic angiogenesis. Nat. Cell Biol. 24, 1726–1738. 10.1038/s41556-022-01026-3 36443426

[B61] Zhao Y. Xue F. Sun J. Guo S. Zhang H. Qiu B. (2014). Genome-wide methylation profiling of the different stages of hepatitis B virus-related hepatocellular carcinoma development in plasma cell-free DNA reveals potential biomarkers for early detection and high-risk monitoring of hepatocellular carcinoma. Clin. Epigenetics 6, 30. 10.1186/1868-7083-6-30 25859288 PMC4391300

[B62] Zhong X. Isharwal S. Naples J. M. Shiff C. Veltri R. W. Shao C. (2013). Hypermethylation of genes detected in urine from Ghanaian adults with bladder pathology associated with Schistosoma haematobium infection. PLoS One 8, e59089. 10.1371/journal.pone.0059089 23527093 PMC3601097

[B63] Ziegler A. Koch A. Krockenberger K. Grosshennig A. (2012). Personalized medicine using DNA biomarkers: a review. Hum. Genet. 131, 1627–1638. 10.1007/s00439-012-1188-9 22752797 PMC3432208

