# Supplementary material for: Exploring TSPAN4 promoter methylation as a diagnostic biomarker for tuberculosis
Source: Front Genet. 2024 Apr 12;15:1380828. doi: 10.3389/fgene.2024.1380828 (PMC11048481; doi:10.3389/fgene.2024.1380828)
Supplement: Supplementary file 2 [file Table3.DOC]

**Table S2**. The detailed information of signature methylation probes.

| **Probes** | **DeltaBeta(Δβ)** | **P.Value** | **P.Val.adj** | **Chromosome** | **Strand** | **Gene** | **Feature** |
| --- | --- | --- | --- | --- | --- | --- | --- |
| cg23213327 | -0.143276504 | 4.06E-07 | 0.001418236 | 2 | R | RSAD2 | TSS1500 |
| cg17984638 | 0.112297247 | 8.94E-06 | 0.002528467 | 4 | R | TXK | TSS200 |
| cg11554335 | -0.133236176 | 1.06E-06 | 0.001688777 | 11 | F | UBE2L6 | TSS1500 |
| cg07839457 | -0.141140432 | 1.32E-04 | 0.007354807 | 16 | R | NLRC5 | TSS1500 |
| cg04552852 | -0.133976641 | 4.60E-08 | 0.000874898 | 11 | R | TSPAN4 | TSS200 |
| cg09313705 | -0.149178336 | 2.05E-07 | 0.001402189 | 17 | R | HOXB2 | TSS200 |
| cg21805118 | 0.099880468 | 3.30E-04 | 0.0123208 | 12 | F | DIABLO | TSS200 |
| cg11804414 | 0.087010899 | 4.46E-04 | 0.014651409 | 12 | F | DIABLO | TSS200 |
| cg19529732 | 0.108379503 | 5.18E-05 | 0.004647847 | 12 | F | DIABLO | TSS200 |
| cg14094409 | 0.110810965 | 1.13E-05 | 0.002664178 | 12 | F | DIABLO | TSS200 |

**Table S3**. The best hyperparameters for machine learning classifiers.

| **Classifier** | **Best parameter** | **Value** |
| --- | --- | --- |
| QDA | reg_param | 0.01 |
|  | tol | 0.0001 |
| LDA | solver | svd |
|  | tol | 0.0001 |
| KNN | leaf_size | 10 |
|  | n_neighbors | 7 |
|  | p | 3 |
|  | weights | distance |
| MLP | activation | logistic |
|  | alpha | 0.0001 |
|  | hidden_layer_sizes | (100,) |
| Logisticregression | penalty | l1 |
|  | C | 10 |
|  | solver | liblinear |
| GaussianNB | var_smoothing | 0.000000001 |
| SVC | probability | TRUE |
|  | C | 31 |
|  | gamma | 0.1 |
|  | degree | 3 |
|  | kernel | rbf |
| Bagging | max_features | 2 |
|  | max_samples | 3 |
|  | n_estimators | 61 |

**Table S4**. The primers and probes for signature methylation probes

| **Method** | **ID** | **Probe** | **Primer Name** | **Primer Sequence** |
| --- | --- | --- | --- | --- |
| Pyrosequencing primer | 1 | cg23213327 | 1.cg23213327-F | GGTTTTTTTTTTTGGTTGGAGTAAT |
|  |  |  | 1.cg23213327-Rbio | ACCCTACAAATCTTAATCCTCCTTAT |
|  |  |  | 1.cg23213327-S | AGTTTTAAAGGTTAGGTTAGT |
|  | 2 | cg17984638 | 2.cg17984638-F | AGTTTAGAGAGGGTAGTTTGATT |
|  |  |  | 2.cg17984638-Rbio | AACTCAATAATCTCCATCCCAATCT |
|  |  |  | 2.cg17984638-S | GTGATTTTTTGAAAATGATGTT |
|  | 3 | cg11554335 | 3.cg11554335-F | TGGGAGTATGAGGAGGGTTATTTTATT |
|  |  |  | 3.cg11554335-Rbio | TTACTCCAATTTCCATAAACACTACC |
|  |  |  | 3.cg11554335-S | AGGGGTAGAGGTTTT |
|  | 4 | cg04552852 | 4.cg04552852-F | GGGGAGATGTATAGAGTTTGAGGTTG |
|  |  |  | 4.cg04552852-Rbio | CTCCAAATCCTAACTTAAACCAAACTAAC |
|  |  |  | 4.cg04552852-S | AAATGGAGGGATAGG |
|  | 5 | cg21805118/cg11804414/  cg19529732/cg14094409 | 5.chr12 122711999-F | TGTTTAGTTTATTTGGGTTTGTGAAATT |
|  |  |  | 5. chr12 122711999-Rbio | AATATAATCTAAAAACCAATTACAACCT |
|  |  |  | 5. chr12 122711999-S | TGGGTTTGTGAAATTATATTT |
| qMSP primer | 6 | ACTB | 6.ACTB COV F | TGGTGATGGAGGAGGTTTAGTAAGT |
|  |  |  | 6.ACTB COV R | AACCAATAAAACCTACTCCTCCCTTAA |
|  |  |  | 6.ACTB COV probe | VIC-ACCACCACCCAACACACAATAA  CAAACACA-MGB-NFQ |
|  | 7 | cg04552852/cg12464638 | 6.TSPAN4 F | ATCCTAACTTAAACCAAACTAACATTCT |
|  |  |  | 6.TSPAN4 R | GGTTCGGGGGAGATGTATAGA |
|  |  |  | 6.ATSPAN4 probe | FAM-AAAATTCCGAATATATAC  GCTTCC-MGB-NFQ |

**Table S5**. Standard plasmid sequence information

| **Plasmid** | **Plasmid Sequence** |
| --- | --- |
| TSPAN4  METH | AAATTTTAATTATGGGTTTTTTTTGGTTTTTTGTTTTGAAGTGATTTTTTTTTTAGTAGGAAGTAGTCGGGGGAGTTTATTTTTTGTTTTTTGTGTTTTGTTTTGATTATAGGAGGTTTTATAACGTTTTTTTTTATAAAATTAGCGTTTTATTTTTTTGGTTTTATTTTAGATTTTTTTTTTTAGAAGTTAGGTCGAGTTTGCGTTAATATTTTATATTTAGGAGAGAGGGGTTTTTATTCGTTTTTGTTGGCGGGTATAGTGTTTTGTTTCGTTATTAGGTTTTTTTTTATTTTCGTTTGTTTGAGGAGGGTTAGGTTTAGGTTTTTTAGGGTTTTTAGGTAGAGTTTAGTCGGGATTGAGGATAGGTTTGGGTTTTTAGGGGTTTTCGTATATATGTGGTGGGGGTTCGGGGGAGATGTATAGAGTTTGAGGTTGGGGTAGTTTTTAGATTTTAGGGGTTTTGTTGGGAAATGGAGGGATAGGAAGCGTATATATTCGGAATTTTAAGAATGTTAGTTTGGTTTAAGTTAGGATTTGGAGAGTAGTTTATTTTTTATTATTTTTTGTTTTGAGTAAATAATTTTTGTAGGTAGGAATAGGGGCGGGATAGGTTTTGGATTTTTTTTTAAGAAGTTTTTAGGTTTTTATTTATTTTTTAGGGTTTATTTTGTTAATATTCGGGATTTTTTTGTTTTTTTTATTTTTATAGTTATTAGTAGTTTCGTAGTTGAATTTGGTTTTGATTTTAGGTTATCGTTATTTAGGGTTTTTGTTTTGTTTTTTTGTTTAGAGTAGGATGTGCGTTTATTTATTTTGGGGGGAGGGGGTTAGGGAAGGGAAGTGGTTTCGGTTCGTGGTTTCGTACGTGGTTGGGTGGGGTATGGTAAATGTTATTGGGGAAGTTGTTTAGGGTTTTGGTTAGGGTGGGAGCGGAAATTTTAAGCGTGTAGTGGATTTGGTTAGTATTATTTCGGATTTAGAAAATTTTTCGTTTTTTT |
| TSPAN4  UNMETH | AAATTTTAATTATGGGTTTTTTTTGGTTTTTTGTTTTGAAGTGATTTTTTTTTTAGTAGGAAGTAGTTGGGGGAGTTTATTTTTTGTTTTTTGTGTTTTGTTTTGATTATAGGAGGTTTTATAATGTTTTTTTTTATAAAATTAGTGTTTTATTTTTTTGGTTTTATTTTAGATTTTTTTTTTTAGAAGTTAGGTTGAGTTTGTGTTAATATTTTATATTTAGGAGAGAGGGGTTTTTATTTGTTTTTGTTGGTGGGTATAGTGTTTTGTTTTGTTATTAGGTTTTTTTTTATTTTTGTTTGTTTGAGGAGGGTTAGGTTTAGGTTTTTTAGGGTTTTTAGGTAGAGTTTAGTTGGGATTGAGGATAGGTTTGGGTTTTTAGGGGTTTTTGTATATATGTGGTGGGGGTTTGGGGGAGATGTATAGAGTTTGAGGTTGGGGTAGTTTTTAGATTTTAGGGGTTTTGTTGGGAAATGGAGGGATAGGAAGTGTATATATTTGGAATTTTAAGAATGTTAGTTTGGTTTAAGTTAGGATTTGGAGAGTAGTTTATTTTTTATTATTTTTTGTTTTGAGTAAATAATTTTTGTAGGTAGGAATAGGGGTGGGATAGGTTTTGGATTTTTTTTTAAGAAGTTTTTAGGTTTTTATTTATTTTTTAGGGTTTATTTTGTTAATATTTGGGATTTTTTTGTTTTTTTTATTTTTATAGTTATTAGTAGTTTTGTAGTTGAATTTGGTTTTGATTTTAGGTTATTGTTATTTAGGGTTTTTGTTTTGTTTTTTTGTTTAGAGTAGGATGTGTGTTTATTTATTTTGGGGGGAGGGGGTTAGGGAAGGGAAGTGGTTTTGGTTTGTGGTTTTGTATGTGGTTGGGTGGGGTATGGTAAATGTTATTGGGGAAGTTGTTTAGGGTTTTGGTTAGGGTGGGAGTGGAAATTTTAAGTGTGTAGTGGATTTGGTTAGTATTATTTTGGATTTAGAAAATTTTTTGTTTTTTT |
