# Supplementary material for: Exploring TSPAN4 promoter methylation as a diagnostic biomarker for tuberculosis
Source: Front Genet. 2024 Apr 12;15:1380828. doi: 10.3389/fgene.2024.1380828 (PMC11048481; doi:10.3389/fgene.2024.1380828)
Supplement: Supplementary file 3 [file Table2.DOC]

**Figure S1.** (A)The soft threshold power and mean connectivity of WGCNA. (B) Dynamic tree cut in WGCNA. (C)The correlation between Module Membership(MM) and Gene Significance(GS) in key module.


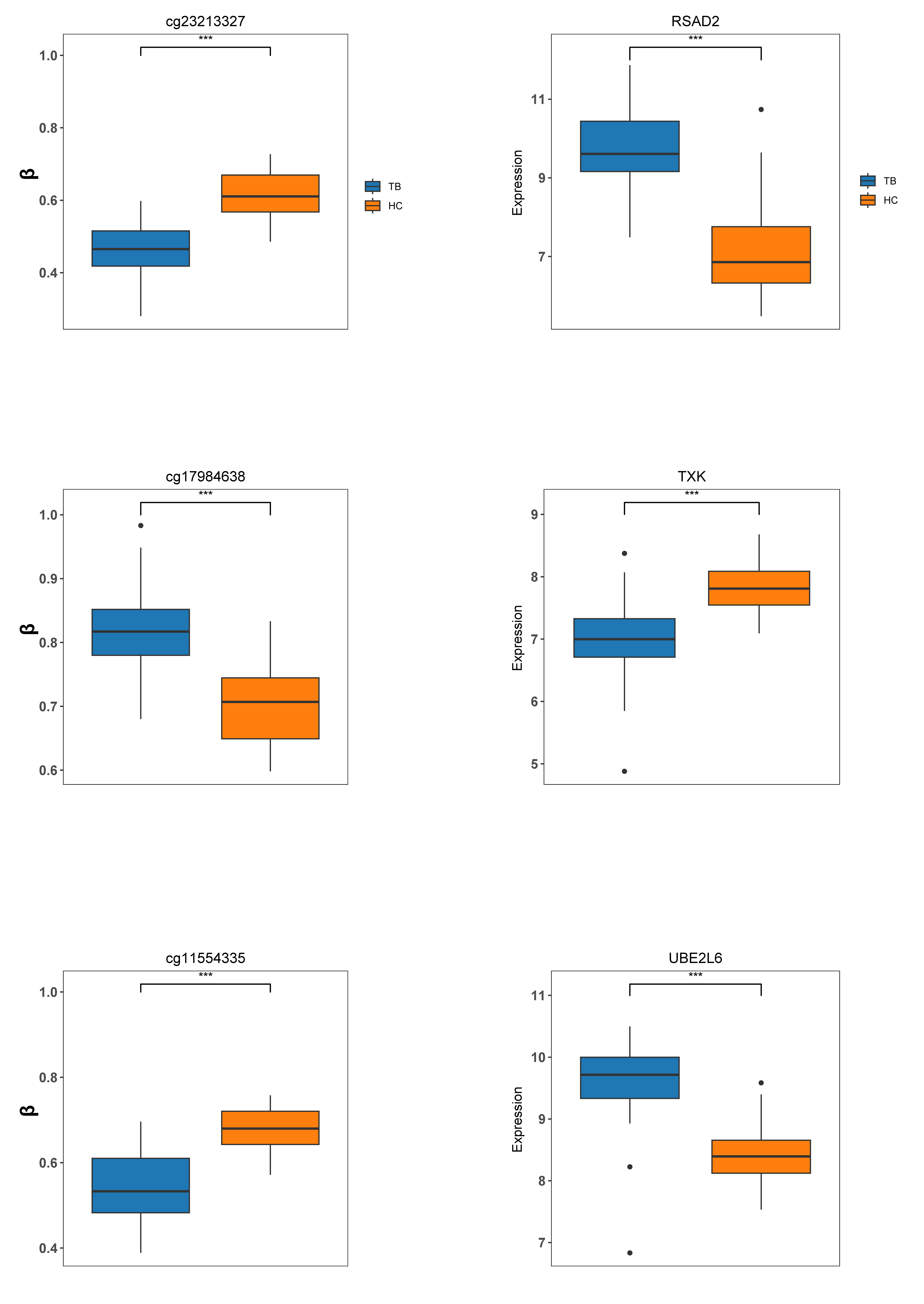


**Figure S2.** Boxplot Comparison of Methylation Positions and Gene Expression





**Figure S3.** Boxplot Comparison of Methylation Positions and Gene Expression


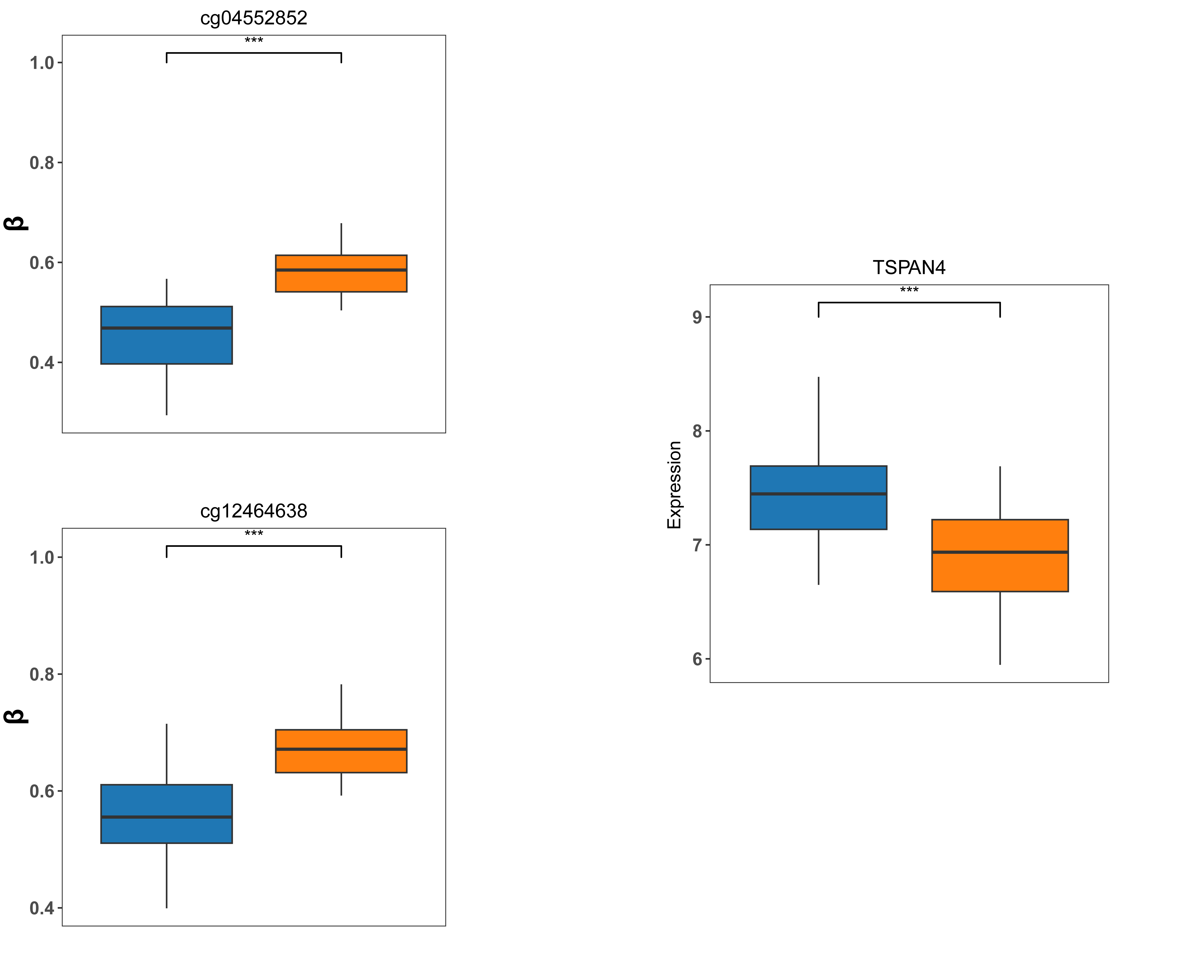


**Figure S4.** Boxplot Comparison of Methylation Positions and Gene Expression
